# A Novel *Coelastrella tenuitheca* Isolate Enhances Growth, Immunity, and Gene Expression in Whiteleg Shrimp

**DOI:** 10.1155/anu/4019255

**Published:** 2025-07-21

**Authors:** Mohamed Ashour, Fawzia S. Ali, Ahmed Mamoon, Mohamed M. Mabrouk, Ahmed I. A. Mansour, Ahmed F. Abdelhamid, Abdallah Tageldein Mansour, Ehab Mohamed, Mostafa Elshobary

**Affiliations:** ^1^Aquaculture Division, National Institute of Oceanography and Fisheries (NIOF), Cairo 11516, Egypt; ^2^Fish Production Department, Faculty of Agriculture, Al-Azhar University, Cairo 11823, Egypt; ^3^Animal and Fish Production Department, College of Agricultural and Food Sciences, King Faisal University, Hofuf, Al-Ahsa, Saudi Arabia; ^4^Department of Integrative Agriculture, College of Agriculture and Veterinary Medicine, United Arab Emirates University, Al Ain 15551, Abu Dhabi, UAE; ^5^Department of Botany and Microbiology, Faculty of Science, Tanta University, Tanta 31527, Egypt

**Keywords:** *Coelastrella terrestris* NIOF17/005, feed additives, gene expression, growth performance, immune status, *Litopenaeus vannamei*, molecular identification

## Abstract

In this study, the freshwater microalga *Coelastrella terrestris* NIOF17/005 was utilized as a feed additive for the Pacific whiteleg shrimp (*Litopenaeus vannamei*). *Coelastrella terrestris* was characterized morphologically, phylogenetically (using 18s rRNA), and biochemically. Shrimp diets were supplemented with *C. terrestris* at 0, 1, 2.5, and 5 g/kg. After a 56-day feeding trial, the biochemical analysis of *C. terrestris* revealed a composition of 38.12% protein, 37.82% lipids, and 15.75% carbohydrates, with valuable bioactive compounds including polyunsaturated fatty acids and antioxidant substrates. The supplementation of 2.5 g/kg *C. terrestris* significantly improved the growth performance of *L. vannamei* (10.23 g final weight), survival rate (92.67%), and feed conversion ratio (1.71). The whole-body composition analysis of *L. vannamei* indicated enhanced protein content (59.74%) and lipid content (7.92%) in the algal-supplemented groups in comparison to the control. Digestive enzyme activities of amylase and lipase increased significantly, with peak activities observed at 2.5 g/kg supplementation (49.55 and 60.06 IU/L, respectively). Nonspecific immunity parameters, including lysozyme (4.47 µg/mL), superoxide dismutase (SOD; 10.77 IU/g), and catalase (CAT; 10.47 IU/g) activities, were substantially elevated in shrimp-fed *C. terrestris*-supplemented diets, with optimal levels at 2.5 g/kg. The gene expression analysis showed that both studied growth-related genes and immunity-related genes were upregulated. These genes reached their maximum expression at a supplementation level of 2.5 g/kg, with the expression levels being approximately 2–3 times higher in the supplemented group in comparison to the control group. In conclusion, these findings suggested that the inclusion of *C. terrestris* NIOF17/005 (2.5 g/kg) into shrimp feed formulations could enhance productivity, improve shrimp health, and potentially increase the sustainability of shrimp farming operations.

## 1. Introduction

In recent decades, aquaculture has emerged as one of the fastest-growing food production sectors worldwide [1, 2]. This expansion has been driven by increasing global seafood demand, coupled with the stagnation of capture fisheries [3, 4]. Within this burgeoning industry, shrimp farming has gained particular prominence, accounting for a significant portion of the aquaculture production and trade [5]. Among commercially farmed shrimp species, *Litopenaeus vannamei* (whiteleg shrimp) is one of the most widely cultivated because of its rapid growth rate and adaptability [6]. *Litopenaeus vannamei* is highly valued for its rapid growth rate, adaptability to a wide range of environmental conditions, and high market demand. The ability of the species to tolerate varying salinity levels and its efficient feed conversion ratio have contributed to its widespread adoption in both coastal and inland aquaculture systems across tropical and subtropical regions [7, 8]. However, intensive farming of *L. vannamei* is challenging [9]. Issues related to nutrition, disease resistance, environmental sustainability, and production costs continue to pose significant hurdles to shrimp farmers and researchers [10, 11]. These challenges have spurred ongoing research on innovative feed formulations, improved culture techniques, and sustainable farming practices to enhance the efficiency and sustainability of *L. vannamei* aquaculture [12].

The interest in microalgae as functional feed additives in aquaculture has indeed been expanding significantly in recent years because of their nutritional profile and bioactive compounds [13, 14]. Microalgae are a treasure chest of several bioactive components including proteins, essential fatty acids, vitamins, minerals, and various bioactive molecules that can potentially enhance the growth criteria, feed consumption, and immune reactions of aquatic organisms [15, 16]. However, the application of locally isolated microalgae strains in aquaculture remains largely unexplored, despite their potential advantages. Local isolates are often better adapted to regional environmental conditions, and may offer unique nutritional profiles tailored to native aquatic species [17, 18]. By focusing on this local isolate, we aim to contribute to the growing body of knowledge on microalgae-based feed supplements, while potentially uncovering a valuable resource for the Egyptian and broader Mediterranean aquaculture industry.

This study introduces a novel isolate of *Coelastrella tenuitheca* from the El-Mahmoudia Canal, Alexandria, Egypt, and marked its first evaluation as an aquafeed additive. Isolation and characterization of local microalgae strains for aquaculture purposes are of paramount importance, as they can lead to the development of region-specific and sustainable feed additives that may outperform commercial and nonnative alternatives [19]. Among the various microalgae genera, *Coelastrella* has gained attention for its potential biotechnological applications and is known for its ability to accumulate high levels of carotenoids and other valuable compounds under stressful conditions [20], making it a promising candidate for use as a feed supplement.

This study aimed to investigate the effects of a novel *Coelastrella* isolate on the growth performance, biochemical composition, digestive enzyme activity, nonspecific immunity, and gene expression of *L. vannamei*. By incorporating different concentrations of the microalgae isolate into shrimp diets, we sought to evaluate its potential as a functional feed additive and its impact on various physiological parameters in cultured shrimp. This study contributes to ongoing efforts to develop sustainable and effective feed strategies for shrimp aquaculture, with a focus on enhancing production efficiency, shrimp health, and overall farm productivity. The findings of this study may provide valuable insights into the application of *Coelastrella*-based feed supplements in *L. vannamei* cultures and their potential benefits for the aquaculture industry.

## 2. Materials and Methods

### 2.1. Microalga Isolate

#### 2.1.1. Water Sampling and Microalgae Strain Isolation

Water samples were collected from a depth of 20 cm below the surface of the El-Mahmoudia Canal in Alexandria, Egypt (31°12'30.07” N, 92°85'66.44” E), located in the northern region of the country. Sterilized jars were used to collect the water samples, which were then quickly transported to the laboratory. In the field, at midday, a 35-cm-diameter Secchi disc was used to monitor the water depth and turbidity, and a mobile temperature/pH meter (Milwaukee MW102) was utilized to record the pH and temperature values. A portable conductivity meter (Oakton, Eutech Instruments) was used to measure salinity. As previously described by Robert [21], the collected water samples were inoculated into BG11 culture medium under controlled temperature and light intensity conditions (25 ± 1°C and 120 μmol photons/m^2^/s, respectively). Following 2 weeks of incubation, 10 mL of healthy colonies were moved and inoculated using the serial dilution method [21] into test tubes that had been sterilized. The culture volume was increased to 10 times, and afterward upscaled to a 20 L volume, under culture conditions of temperature (25 ± 1°C), light intensity of 120 μmol photons/m^2^/s, photoperiod of 16:8 h (light:dark), and pH 7.5. When the cultures reached the late exponential phase (LEP), after 10 days, the cultures were centrifuged (4000 × *g* for 10 min), and the supernatants were discarded. Microalgal pellets were collected and oven-dried at 40°C for 48 h to determine the algal biomass at LEP. Morphological investigations of the isolated strain were conducted using standard classification key references [22, 23] and a light binocular microscope (Olympus BX51 light microscope, Tokyo, Japan). The molecular classification method was used to confirm the findings derived from the morphological investigations of the isolates.

#### 2.1.2. Phylogenetic Identification

Genomic DNA of the isolated microalga was extracted using a modified CTAB method [24]. A partial sequence of the 18s rRNA gene was amplified by PCR, using universal primers and a specific thermal cycling protocol [25, 26]. The DNA analysis procedure involved multiple sequential steps as follows: PCR products were initially visualized on agarose gel, then purified with the QIAquick Gel Extraction Kit (Qiagen, Germany), and subsequently sequenced using an Applied Biosystems 3730xl DNA Analyzer (Thermo Fisher Scientific, USA). The resulting 18s rDNA sequences underwent comparative analysis against GenBank entries using BLAST (NCBI), followed by multiple sequence alignment performed with the MUSCLE algorithm implemented in MEGA11 software to determine phylogenetic relationships [27], and a neighbor-joining phylogenetic tree was constructed to identify closely related species and assess water quality.

#### 2.1.3. Biochemical Compositions

To determine the biochemical content (total proteins, carbohydrates, and lipids), 100 mL of the cultured isolated strain was centrifuged (4000 × *g* for 10 min). After centrifugation, the pellet was stored at −20°C, and the supernatant was discarded. Total protein was extracted using the procedure outlined by Rausch [28], and the quantity of total proteins was determined using the Hartree [29] procedure. The process described by Myklestad and Haug [30] was followed to extract the total carbohydrates, and the method by DuBois et al. [31] was used to measure their quantity. Total lipid levels were measured according to the procedure described by Bligh and Dyer [32].

#### 2.1.4. GC-Mass Analysis

To determine the biologically active materials of the microalgae isolate, a methanolic extract of the isolate dry weight (0.1 g) was conducted using methanol 70%. About 1 mL of the extract was used to assess the biologically bioactive compounds of the studied isolate using chromatography–mass spectrometry (GS–MS). The mass spectrometer was configured to operate at 70 electron volts (eV) and examined fragments ranging from 50 to 650 *m*/*z*. Peaks in the crude extracts were identified by comparing the obtained mass spectra with those in the NIST library.

### 2.2. Shrimp Feeding Experiment

#### 2.2.1. White Leg Shrimp *Litopenaeus vannamei*

From a commercial hatchery near Kafr El-Sheikh City, the postlarvae (PLs) of *L. vannamei* were safely transferred to the Fish Nutrition Laboratory at Baltim Research Station, Alexandria Branch of NIOF. The PLs were placed in 5 m × 5 m × 1 m concrete tanks for 15 days to allow them to acclimate to the surrounding laboratory experimental conditions. The tanks were maintained at a water temperature of 25 ± 2°C, salinity of 26 ± 1 ppt, and constant aeration to keep a dissolved oxygen (DO) content of 5 mg/L. During the adaptation period, PLs were fed a traditional commercial diet four times per day (Aller Aqua, Giza Governorate, Egypt).

#### 2.2.2. Shrimp Experimental Conditions

In this feeding trial, the 600 PLs were divided into four groups. After two weeks of acclimatization, 150 PLs per group were randomly allocated into three replicates per group (50 PLs per replicate). The shrimp experiment was conducted in net hapas (0.7 m × 0.7 m × 1 m), fixed in concrete ponds (4 m × 2 m × 1 m). Every pond had a daily water exchange of about 10% owing to the input and output flow rates throughout the pond. Each hapas net was cleaned daily during the experiments. During the eighth week, the PLs were kept under a temperature of 26 ± 2°C, salinity of 26 ± 1 ppt, photoperiod of 12:12 h, and DO of 5.5 ± 1 mg/L by constant aeration. The levels of NH_3_, NO_2_, NO_3_, and pH were observed following the protocol previously recommended by APHA [33], and the reported values (0.08 ± 0.01, 0.10 ± 0.01, 0.18 ± 0.02, and 7.70 ± 0.20 mg/L, respectively) seemed to be within the acceptable range for shrimp cultivation [7].

#### 2.2.3. Experimental Diets

PLs were fed, for 8 weeks in one of the four dietary groups; D_0_, a commercial shrimp diet (Aller-Aqua, Giza Governorate, Egypt), which served as a control diet with the following nutritional values: 45% protein, 8% fat, 34% carbohydrates, 3.65% fiber, and 9% ash. Dry powder of the microalgae isolate was added to the diets, as previously reported by Ashour et al. [34]. In brief, the diet was powdered and divided into three groups. Microalgae powder (at concentrations of 1, 2.5, and 5 g/kg diet for D_1_, D_2_, and D_3_, respectively) was dissolved in a low volume of distilled water. Microalgae were sprayed and applied as surface coatings to ensure their adhesion to the pellets. The control diet (D_0_) was sprayed with equal amounts of distilled water without the microalgae. Then, 5 cm of sunflower oil per kg of diet was sprayed onto the pellets after drying at 40°C for 48 h, achieving a final moisture content of less than 10%. The diets were stored at 4°C until use.

### 2.3. Shrimp Investigated Parameters

#### 2.3.1. Growth, Survival, and Nutrient Utilization

To calculate weight gain (WG, g), the initial weight (IW, 1.66 ± 0.12 g per PL), and final weight (FW, g) of the PLs were recorded at the beginning and end of the experiment, respectively. The survival rate, feed conversion ratio, specific growth rate, feed efficiency ratio, and protein efficiency ratio were determined using the following equations.

(1) $$\begin{array}{c} \text{Weight gain}\left(\text{WG},\text{g}\right)=\text{Final body weight}\left(\text{g}\right)-\text{initial body weight}\left(\text{g}\right). \end{array}$$

(2) $$\begin{array}{c} \text{Survvival rate}\left(\text{SR},\%\right)=\left(\text{The total final survived number of shrimp}/\text{the initial number of shrimp}\right)\times 100. \end{array}$$

(3) $$\begin{array}{c} \text{Specific growth rate}\left(\text{SGR}\%/\text{day}\right)=\left(\left(\text{Ln final body weight}-\text{Ln initial body weight}\right)/\text{t}\right)\times 100. \end{array}$$

(4) $$\begin{array}{c} \text{Feed conversion ratio}\left(\text{FCR}\right)=\text{Total consumed feed}/\text{WG}. \end{array}$$

(5) $$\begin{array}{c} \text{Feed efficiency ratio}\left(\text{FER}\right)=\left(\text{WG}\left(\text{g}\right)\right)/\left(\text{feed intake}\left(\text{g}\right)\right). \end{array}$$

(6) $$\begin{array}{c} \text{Protein efficiency ratio}\left(\text{PER}\right)=\left(\text{WG}\left(\text{g}\right)\right)/\left(\text{protein intake}\left(\text{g}\right)\right). \end{array}$$

#### 2.3.2. Biochemical Composition

Dry matter, total crude lipid, total crude protein, and ash contents were assessed following the standard procedures previously outlined by the AOAC [35]. Five PL samples were taken from each replication when the experiment ended to examine the biochemical constitution. Before evaluation, the PLs random samples were combined, dried, ground, and kept at a cool temperature (–20°C).

#### 2.3.3. Digestive Enzymes

The shrimp were fasted for 24 h, euthanized by decapitation, and dissected to collect tissue for analysis. To determine the activities of the digestive enzymes (lipase and amylase), homogenized gastrointestinal glands of the six samples/treatments were collected and centrifuged. The calculations of digestive enzyme properties were conducted using a spectrophotometric approach with specific kits following the manufacturer's instructions (Biodiagnostic Co., Egypt, Cat No. 281001 and AY1050 for lipase and amylase, respectively). Lipase and amylase levels were measured at wavelengths of 580 and 660 nm, respectively [10].

#### 2.3.4. Nonspecific Immunity and Antioxidant Status

For nonspecific immunity status, six euthanized shrimp samples were randomly selected and frozen at −80°C until analysis. Homogenized samples were exposed to a pH of 7.4 in LBS and centrifuged for 20 min at 3000 rpm. Subsequently, the antioxidant and lysozyme were measured. Lysozyme (Lys) ELISA Kits (Cat No.: SL0050FI, SunLong Biotech Co., Ltd, China). *Micrococcus lysodeikticus* cells incubated with lysozyme were used as a medium for the assay. The reaction was monitored by measuring the drop in absorbance at 450 nm according to the manufacturer's instructions [36].

The antioxidant properties of MDA (lipid peroxide, malondialdehyde), superoxide dismutase (SOD), and CAT (catalase) were conducted using the colourimetric approach. Particular kits for MDA (Cat NO.: MD2529), SOD (Cat NO.: SD2521), and CAT (Cat NO.: CA2517) were obtained from the Biodiagnostic Company, Egypt, and the measurement methods were following the manufacturer's instructions. Using the spectrophotometric method, wavelengths of 534, 560, and 510 nm were applied to MDA [37], SOD [38], and CAT [39], respectively.

#### 2.3.5. Impact of Microalgae Isolate on Shrimp's Genes Expressions

After completing the experiment, six shrimp were collected from each group. Total RNA was purified from muscle tissues utilizing Easy-RED from INTRON Korea, based on the information provided with the kit. The quantitative yield and purity of isolated RNA were assessed using a NanoDrop device (Implen, Nanophotometer, NP80 touch, Germany). The isolated RNAs were used as a template for synthesizing cDNAs. Some shrimp genes related to growth and immunity (three genes for each) were tested for their expression by cDNA amplification using RotorGene Q thermal cycling. The growth-related genes, included growth hormone (*GH*), insulin-like growth factor-I (*IGF-I*), and insulin growth factor-II (*IGF-II*). These genes play crucial roles in regulating growth, development, and metabolic processes in shrimp. Immunity-related genes comprised lysozyme (*Lys*), prophenoloxidase (*proPO*), and *SOD* (an antioxidant gene). Primers used for the amplification of the target genes were described by [34, 40]. Each qPCR run was done using 20 μL of the low ROX SYBR Green kit, 4 μL (50 ng) of cDNA, and 0.500 ng of each primer 10 μM, and 5 μL water (RNAse-free) were used in the test. The applied thermal profile followed Hassan et al. [40]. The temperature was gradually increased by 0.5°C from 60 to 95°C, allowing the melting curve for the target gene products to be obtained. β-actin served as the reference gene for these reactions [40]. The amplicon sizes and primer sequences used are listed in Table 1. Using the 2^-*ΔΔ*Ct^ equation [41], the β-actin housekeeping gene was used to calculate the *ΔΔ*Ct values of target genes [25].

All relevant institutional, national, and international laws pertaining to the use of animals were followed when conducting this study. The National Institute of Oceanography and Fisheries (NIOF) Committee for Ethical Care and Use of Animals/Aquatic Animals (NIOF-IACUC) carefully reviewed the research protocol for this work.

### 2.4. Statistical Analysis

Before conducting this analysis, assumptions of normality and homogeneity were checked using Levene's test [42]. The results in percentage form were transformed using arcsine based on the method recommended by Zar [43]. The findings of the shrimp study are expressed as the mean ± SD. Data analyses were conducted by employing the SPSS 21 Statistics Software with the tools of one-way ANOVA and the subsequent Duncan [44] test, with a significance level of *p* ≤ 0.05. The figures were graphically presented using GraphPad (Prism 8) Statistics Software.

## 3. Results and Discussion

### 3.1. Freshwater Microalga Isolate (*Coelastrella tenuitheca* NIOF17/005)

#### 3.1.1. Morphological and Scanning Electron Microscopy (SEM) Characterizations

**Figure 1:**
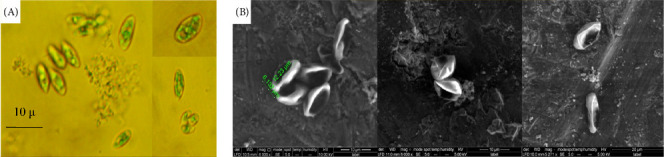
Light (A, 400x) and scanning electron microscope, (B) observations of the freshwater microalgae isolate *Coelastrella terrestris* NIOF17/005.

Morphological and SEM characterization (Figure 1A,B) were used to elucidate the structural features of the isolated freshwater microalgae (*C. tenuitheca*).

Light microscopy revealed spherical to oval-shaped cells, typically ranging from to 5–20 μm in diameter, with a smooth cell wall characteristic of the genus [45]. Asexual reproduction through autospore formation was observed, which was consistent with other Coelastrella species (Figure 1A). SEM provided detailed insights into the cell wall ultrastructure, revealing subtle surface textures that were not discernible under light microscopy [46]. The presence and nature of extracellular polymeric substances (EPSs) were also examined using SEM (Figure 1B). These combined morphological and ultrastructural observations contribute to a comprehensive understanding of the cellular architecture of *C. tenuitheca* and provide valuable taxonomic information to distinguish this species within the genus *Coelastrella*.

#### 3.1.2. Phylogenetic Identification

**Figure 2:**
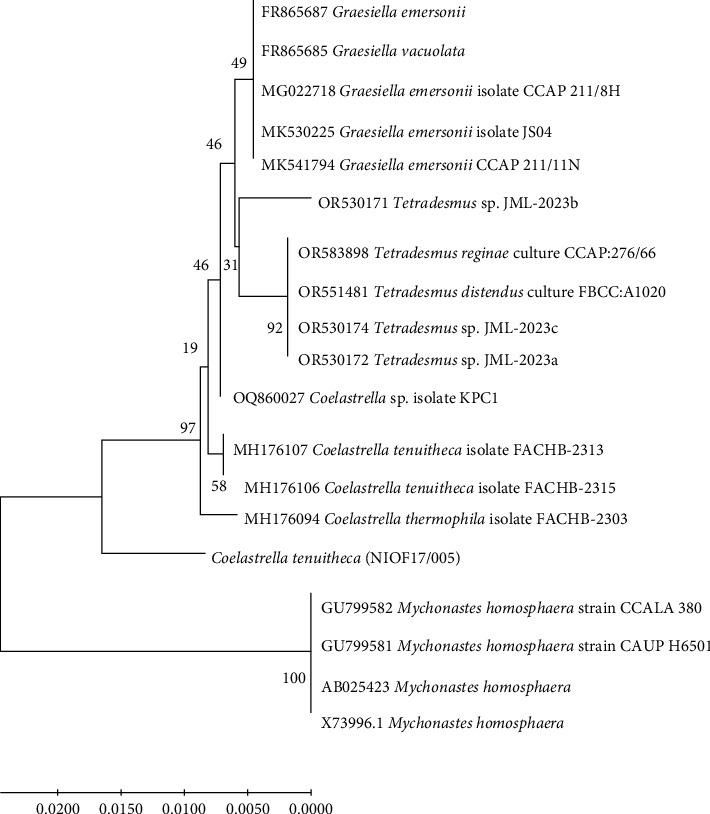
Neighbor-joining (NJ) tree based identification of the isolated microalgal strain based on 18s rRNA nucleotide sequences (the tree's branches display the bootstrap values).

Phylogenetic analysis (Figure 2) strongly supported the classification of the studied isolate within the genus *Coelastrella*. Specifically, the isolate showed 98% identity with *Coelastrella terrestris*, suggesting that it belonged to this species. The sequence was subsequently submitted to GenBank under the Accession Number PQ164244.

The *Coelastrella* clade in the constructed phylogenetic tree was distinctly separated from other genera, including *Scenedesmus*, *Desmodesmus*, and *Mychonastes* [47]. This molecular evidence corroborates and refines our initial morphological identification and provides a robust basis for taxonomic classification. The phylogenetic tree topology and branch lengths indicate the degree of genetic similarity between the studied isolate and related species, offering valuable insights into the evolutionary relationships within the green algae lineage [46].

#### 3.1.3. Biochemical Composition of *Coelastrella terrestris* NIOF17/005

Table 2 shows the biochemical components (proteins, lipids, and carbohydrates) of the freshwater isolate *C. terrestris* NIOF17/005 at the LEP. The mean dry weight of *C. terrestris* NIOF17/005 at LEP was 0.89 ± 1.61 g/L. The averages of total protein, fat, and carbohydrate, as a percentage of dry weight, were 38.12% ± 1.61%, 37.82% ± 1.80%, and 15.75% ± 2.05%, respectively. Saadaoui et al. [48] screened 30 local freshwater microalgae isolates based on growth rate to determine the best strains that can be utilized as feed for livestock. A normality test was used to select 15 fast-growing microalgal strains among these isolates for further examination of their metabolite content. The screening method identified *Mychonastes homosphaera* QUCCCM70 as a highly nutritious strain among all examined strains. They concluded that this strain has the potential to generate a well-balanced animal feed supplement to improve the quality of livestock including poultry and cattle. This strain contained 37.3% protein, 40.7% fat, and 12.4% carbohydrate. In the same line with the findings reported by Saadaoui et al. [48], the present study reported that the biochemical composition exhibited by *C. terrestris* strain NIOF17/005 candidates this strain to be an attractive species to be utilized as a dietary feed *supplement* for shrimp *L. vannamei*.

#### 3.1.4. GC–Mass Chromatogram Analysis

**Figure 3:**
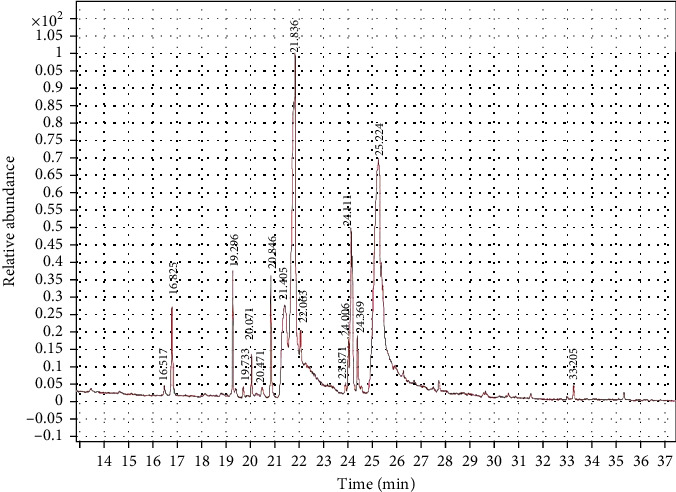
GC–MS chromatogram of *Coelastrella terrestris* NIOF17/005.

The GC–MS findings for *C. terrestris* strain NIOF17/005 showed 16 retention times based on the peak areas (PAs; Figure 3). As presented in Table 3, the 16 phytochemical compounds belonged to seven phytochemical groups. The most abundant group was fatty acids (two RT with 54.64% of the total PA) and consisted of two polyunsaturated fatty acids, linoleic acid, and eicosapentaenoic acid (EPA). Linoleic acid is a high polyunsaturated fatty acid (C18:2) and belongs to omega-6 FA, as previously reported by Hur et al. [62], this FA exhibited highly potent antioxidant activities. EPA is a highly polyunsaturated fatty acid (C22:5) that belongs to the omega-3 FA. FA is very important for aquatic animals, is considered an immunity and growth enhancer, and has previously shown highly potent antioxidant activities [56]. The second major group was the oil-soluble vitamin C derivative (one RT with 23.87% of the total PA), represented by l-(+)-ascorbic acid 2,6-dihexadecanoate (ascorbic acid dipalmitate). Gopinath et al. [57] reported that ascorbic acid dipalmitate exhibits highly potent antioxidant activity.

The third major group was fatty acid esters (eight RT with 10.82% of total PA) and consisted of four fatty acid methyl esters (FAMEs), which contributed 10.82% of total PA; oleic acid, methyl ester (7.61%), palmitic acid, methyl ester (3.21%), and 9,12-octadecadienoic acid, (2-phenyl-1,3-dioxolan-4-yl) methyl ester, cis-(trace less than 1% of total PA); and 8,11-octadecadienoic acid, methyl ester (trace). The other four FA esters were found in trace amounts (less than 1% of the total PA). Besides the FAMEs, there are two fatty acid propanetriyl esters; 1.9,12-octadecadienoic acid (Z,Z)-, 1,2,3-propanetriyl ester (Trilinolein), and Docosahexaenoic acid, 1,2,3-propanetriyl ester (a polyunsaturated omega-3 fatty acid, C22:6, DHA), one fatty acid ethyl ester (FAEE); hexadecanoic acid, ethyl ester, and one fatty acid eicosyl ester; oleic acid, eicosyl ester.

The fourth and fifth major groups were alcoholic and alkane compounds, respectively (one RT each with 4.22% and 1.77% of the total PA, respectively). It is present in ethanol, 2-(9,12-octadecadienyloxy)-, (Z, Z)-, and nonadecane. As previously reported, nonadecane, a bioactive material in seaweed extract, improved the immunity and growth of shrimp [64]. Khan et al. [51] reported that the leaf extract of the medicinal plant fernleaf yarrow, *Achillea filipendulina* (L.), which contains octadecadienyloxy, showed novel, safe, and highly potent antioxidant properties. The remaining three bioactive compounds, represented as traces, belong to two phytochemical groups; the carotene group (two RTs and two compounds: lycopene and lycopene-16-ol) and the steroid group (one RT with one compound of Cholestane-3-ol, 2-methylene-, (3*β*,5*α*)-, and the saturated tetracyclic triterpene). Carotene compounds are well-known as strong antioxidant materials [49, 59], whereas cholestane compounds are also well-known to have strong antioxidant activities [61].

### 3.2. Shrimp

#### 3.2.1. Survival, Growth, and Nutrient Consumption Efficiency

Table 4 shows the survival, growth, and nutrient consumption parameters of shrimps supplemented with the freshwater microalgae isolate *C. terrestris* NIOF17/005. Compared with the control group (D_0_), significant (*p* < 0.05) improvements were observed in all experimental growth (FW, WG, DWG, and SGR), nutrient utilization efficiency (FCR, FER, and PER), and SR values in all groups supplemented with the freshwater microalgae isolate *C. terrestris* NIOF17/005 (D_1_, D_2_, and D_3_). These increases in values improved with an increase in *C. terrestris* NIOF17/005 dietary supplementation levels up to 2.5 g/kg diet (D_2_).

**Figure 4:**
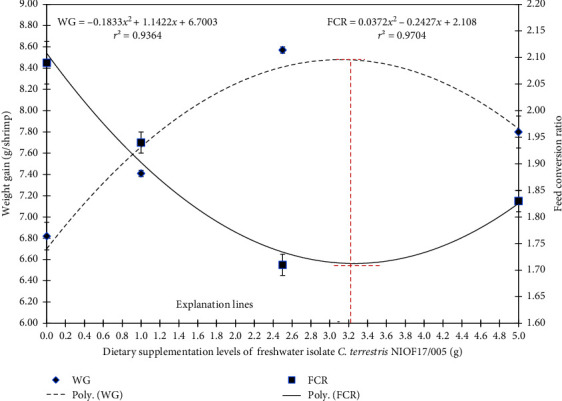
Polynomial regression of shrimp weight gain, dietary supplementation levels (g), and feed conversion ratio of the freshwater isolate *Coelastrella terrestris* NIOF17/005.

Figure 4 illustrates the polynomial regression of FCR and WG, showing that with the increase in dietary supplementation of *C. terrestris* NIOF17/005, the WG polynomial regression improved (*r*^2^ = 0.9364), while the FCR polynomial regression reduced (*r*^2^ = 0.9704). Based on the literature, this is the first study to investigate the possible application of the green microalga isolate *C. terrestris* as feed additives for *L. vannamei*. The obtained results were in the same line of findings reported by Sharawy et al. [65] who concluded that the nanoparticle application of blue–green alga, *A. platensis* as an aquafeed additive significantly improved the growth performance, survival, and feed consumption efficiency of shrimp for *L. vannamei*. These improvements can be attributed to the unique bioactive materials in this isolate, especially the FA content, which enhanced the growth and development of *L. vannamei*.

Based on the data illustrated in Figure 4, the explanation lines of the highest and lowest peaks of FCR and WG, as shown in the polynomial regression model, predict that the broken line with the ideal inclusion level of freshwater isolate *C. terrestris* NIOF17/005 is 3.12 g/kg diet. This ideal range inclusion level exists in group D_2_ (2.5 g/kg).

#### 3.2.2. Shrimp Whole-Body Carcass Constituents

Table 5 illustrates the whole-body carcass constituents of *L. vannamei* fed diets supplemented with different concentrations (0, 1, 2.5, and 5 g/kg diet) of the freshwater microalgae isolate *C. terrestris* NIOF17/005.

As reported in Table 5, there were significant (*p* < 0.05) improvements in dry mass, protein, lipid, and ash content of *L. vannamei* in the supplemented groups (D_1_, D_2_, and D_3_) compared to the control group (D_0_). The carcass percentage values of dry matter and fat increased with an increase in *C. terrestris* concentration up to 5 g/kg diet (D_3_). While protein and ash percentage values were increased with the increase of *C. terrestris* concentration up to 2.5 g/kg diet (D_2_). This may be attributed to the high biochemical composition of *C. terrestris* NIOF17/005, which represents, as illustrated in Table 2, lipids of 37.82%, proteins of 38.12%, and carbohydrates of 15.75%. In the same line as our findings, the study conducted by Dimitrova et al. [66] stated that the protein, lipid, and carbohydrate contents of *Coelastrella* sp. cultured under different temperatures (20–44 °C) and illumination (8000 and 16,000 lx) range between 18.3%–57.9%, 7.3%–50.7%, and 11.8%–50.7%, respectively. Saadaoui et al. [48] concluded that the *M. homosphaera* strain QUCCCM70 consisted of 37.3% protein, 40.7% fat, and 12.4% carbohydrate, indicating that this strain exhibited qualified biochemical composition and candidates as an attractive species to be utilized as a dietary feed additive in aquaculture.

#### 3.2.3. Digestive Enzymes and Nonspecific Immunity Properties

Table 6 illustrates the digestive enzymes, antioxidant activities, and nonspecific immunity properties of *L. vannamei* fed with several doses of the freshwater microalgae isolate *C. terrestris* NIOF17/005.

In the current study, compared to D_0_, shrimp in the supplementation groups (D_1_, D_2_, and D_3_) exhibited significant (*p* < 0.05) improvements in the activities of the digestive enzymes (amylase and lipase) and nonspecific immunity of lysozyme, SOD, and CAT activities, while no significant (*p* < 0.05) differences in MDA activity were observed between the experimental groups (Table 6). Jerez-Cepa and Ruiz-Jarabo [67] concluded that immune-correlated factors are considered a predicted indication of aquatic animals' health status. Owing to the lack of adaptive immunity, shrimp mostly depend on nonspecific immunological processes [68]. Lysozyme and SOD enzymes destroy bacteria and scavenge free radicals [69]. MDA is typically used as an oxidative stress marker because it indicates increased free radical generation [70]. Humoral substances, such as lysozyme, SOD, CAT, and MDA play a crucial role in shrimp immunity and pathogen/nonedogenous substance removal [16]. Amylase and lipase activities increased with an increase in *C. terrestris* concentration up to 5 g/kg diet (D_3_). While lysozyme, SOD, and CAT activities were increased with the increase of *C. terrestris* concentration up to 2.5 g/kg diet (D_2_). Our results were confirmed previously by Xu et al. [71] who stated that the powerful phytochemical compounds of microalgae could enhance the release of digestive enzymes and consequently enhance feed digestibility and absorption. Similarly, several studies concluded that the addition of microalgae in shrimp diets led to a significant improvement in nonspecific immunity. Ashour et al. [16] documented that the nonspecific immunomodulatory activities of *L. vannamei* were significantly enhanced in the case of feeding on dietary supplementation with several concentrations of the microalgae *A. platensis*, compared to the control basal diet.

#### 3.2.4. Genes Expressions

**Figure 5:**
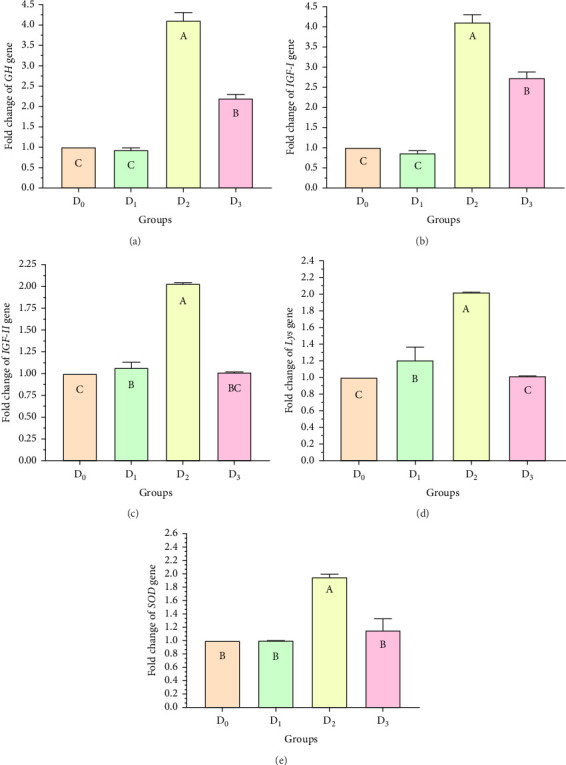
(a–e) Growth-related genes (*GH*, *IGF-1*, *IGF-II*), immunity-related genes (*Lys*, *proPO*, and *SOD* antioxidant gene expressed at different levels in the muscle tissues of *L. vannamei* (*n = 3*) that were fed diets containing different doses of the freshwater microalgae isolate *Coelastrella terrestris* NIOF17/005. Significant differences are indicated by columns with different capital letters (*p* < 0.05).

Figure 5 illustrates the expression of growth-associated genes (*GH*, *IGF-1*, and *IGF-II*) and immunity-related genes (*Lys*, *ProPO*, and *SOD*) in *L. vannamei* fed on diets including different levels of freshwater isolate *C. terrestris* NIOF17/005. Regarding the studied genes in the current work, there were three growth (*GH*, *IGF-1*, and *IGF-II*) and immunity (*Lys*, *ProPO*, and *SOD*) related genes. GH, which is synthesized in the pituitary gland, and works on target cells to promote the growth of shrimp and aquatic animals [72]. *IGF-I* is an important peptide hormone that regulates shrimp's development and growth [73].

*IGF-II* is another important protein of the *IGF* family, contributing to fetal growth and development, as well as aiding in tissue repair and regeneration in adult shrimps [74]. *Lys* is an antimicrobial enzyme that plays a pivotal role in the shrimp's immune defense system owing to its presence in the hemolymph. This versatile enzyme effectively combats a broad spectrum of bacteria, protecting against bacterial infections [75]. The ProPO-activating system is one of the most important nonspecific immune responses in crustaceans like shrimp, as they lack adaptive immunity. Upon encountering pathogens, proPO is activated and initiates melanization [76]. SOD is an antioxidant enzyme crucial for protecting shrimp cells from harm. By converting superoxide radicals into less harmful molecules, SOD maintains cellular functionality and protects the shrimp from harmful free radicals [77].

Figure 5 shows that the supplemented diets provided significantly better results (*p* < 0.05) than the control diet (D_0_). In contrast, D_3_ provided significantly superior results (*p* < 0.05) than D_2_ and D_4_. Moreover, the results presented that the expression of both growth factors (Figure 5), and immunity-related genes (Figure 5) increased with increasing *C. terrestris* dietary inclusion levels up to 2.5 g/kg diet (D_2_), and gene expression was decreased in the 5 g/kg diet (D_3_). Previous studies agree with the current findings, indicating that incorporating microalgae into the diet improves the regulation of growth- and immune-related genes in the shrimp [16] and Nile tilapia [78].

## 4. Conclusions

This study displays the significant potential of isolated *Coelastrella terrestris* NIOF17/005 as a valuable feed additive (chemical composition and active components) in shrimp aquaculture of *Litopenaeus vannamei*. The 56-day feeding trial revealed that supplementation with *C. terrestris*, particularly at 2.5 g/kg, led to substantial improvements in the growth performance, survival rates, and feed utilization efficiency of *L. vannamei*. Enhanced digestive enzyme activities (amylase and lipase) and elevated nonspecific immunity parameters (lysozyme, SOD, and CAT) were observed, indicating improved digestive efficiency and boosted innate immune responses. The upregulation of both growth-related and immunity-related genes provides molecular evidence supporting the reported physiological improvements. These findings suggest that the integration of *C. terrestris* NIOF17/005 into shrimp feed formulations could enhance productivity, improve shrimp health, and potentially increase the sustainability of shrimp farming operations. Future research should focus on long-term studies, potential synergistic effects with other feed additives, economic analysis, and impacts on product quality to further validate the application of *C. terrestris* in commercial shrimp farming.

## Figures and Tables

**Table 1:** Quantitative real-time PCR primers used for gene expression analysis.

Primer codes and accession numbers	Sequences (5′→3′)	Amplicon size (bp)
*GH* (XM027360152)	F: AATTTGCGCTTGCACTACGG R: ATCCGGTTGAGGTGTAGCTG	100

*IGF-I* (KP420228)	F: GTGGGCAGGGACCAAATC R: TCAGTTACCACCAGCGATT	123

*IGF-II* (XM027379465.1)	F: CTCTGTACAGTCAGCCCAGC R: CACACCCAGTCAGTCCCAAG	220

*Lys* (XM_027352840.1)	F: GGACTACGGCATCTTCCAGA R: ATCGGACATCAGATCGGAAC	97

*proPO (*XM_027379995.1)	F: CGGTGACAAAGTTCCTCTTC R: GCAGGTCGCCGTAGTAAG	122

*SOD* (DQ005531)	F: AATTGGAGTGAAAGGCTCTGGCT R: ACGGAGGTTCTTGTACTGAAGGT	153

β-actin (AF300705)	F: GCCCATCTACGAGGGATA R: GGTGGTCGTGAAGGTGTAA	121

Abbreviations: GH, growth hormone; IGF-I, insulin-growth factor-I; IGF-II, insulin growth factor-II; Lys, lysozyme; proPO, prophenoloxidase; SOD, superoxide dismutase.

**Table 2:** Biochemical composition analysis (% of dry biomass) of *Coelastrella terrestris* NIOF17/005.

Biochemical compositions (based on dry weight, %)	Values
Dry weight (g/L)	0.89 ± 0.61
Total protein (%)	38.12 ± 1.61
Total lipid (%)	37.82 ± 1.80
Total carbohydrates (%)	15.75 ± 2.05

**Table 3:** Bioactive compounds reported in freshwater isolate *Coelastrella terrestris* NIOF17/005*⁣*^*∗*^.

RT	PA%	Compound name	Formula	Molecular weight	Nature	Biological activities	References
16.517	T^a^	Lycoxanthin (lycopene-16-ol)	C_40_H_56_O	552.43	Carotene	Antioxidant	[49]
16.60	T^a^	Nonadecane	C_19_H_40_	268.31	Alkane compound	Growth enhancer, antioxidant	[50]
16.82	2.77	Ethanol, 2-(9,12-octadecadienyloxy)-, (Z,Z)-	C_20_H_38_O_2_	310.29	Alcoholic compound	Antimicrobial	[51]
19.30	4.12	1.9,12-octadecadienoic acid (Z,Z)-, 1,2,3-propanetriyl ester (trilinolein)	C_57_H_98_O_6_	878.73	Fatty acid propanetriyl ester (triacylglycerol)	Antioxidant	[52]
19.73	T	Docosahexaenoic acid, 1,2,3-propanetriyl ester	C_69_H_98_O_6_	1022.74	Fatty acid propanetriyl ester (DHA)	Anticholesterol compound	[53]
20.07	T	9,12-octadecadienoic acid, (2-phenyl-1,3-dioxolan-4-yl)methyl ester, cis-	C_28_H_42_O_4_	442.39	FAME	Antioxidant, antimicrobial	[26, 53]
20.47	T	Palmitic acid, methyl ester	C_17_H_34_O_2_	270.26	FAME	Anti-contractile, anti-inflammatory	[54, 55]
20.84	3.21	cis-5,8,11,14,17-eicosapentaenoic acid	C_20_H_30_O_2_	302.22	Fatty acid (EPA)	Immunity and growth enhancers	[56]
21.40	8.16	l-(+)-Ascorbic acid 2,6-dihexadecanoate (ascorbic acid dipalmitate)	C_38_H_68_O_8_	652.49	Oil-soluble vitamin C derivative	Antioxidant	[57]
21.84	23.86	Hexadecanoic acid, ethyl ester	C_18_H_36_O_2_	284.27	FAME	Antioxidant, antimicrobial	[58]
22.06	T	Lycopene	C_40_H_56_	536.44	Carotene	Antioxidant, inflammatory	[49, 59]
23.87	T	8,11-Octadecadienoic acid, methyl ester	C_19_H_34_O_2_	294.26	FAME	Growth enhancer, antioxidant	[50 ]
24.01	T	Oleic acid, methyl ester	C_19_H_36_O_2_	296.27	FAME	Antioxidant, antimicrobial	[60]
24.11	7.614	Cholestan-3-ol, 2-methylene-, (3β,5α)-	C_28_H_48_O	400.37	Steroid (saturated tetracyclic triterpene)	Antioxidant, antimicrobial	[61]
24.37	T	Linoleic acid	C_18_H_32_O_2_	280.24	Fatty acid	Antioxidant	[62]
25.22	46.49	Oleic acid, eicosyl ester	C_38_H_74_O_2_	562.57	Fatty acid eicosyl ester	Antimicrobial	[63]

Abbreviations: FAEE, fatty acid ethyl ester; FAME, fatty acid methyl ester; PA, peak area; RT, retention time.

^a^Percentages less than 1% traces.

**Table 4:** Growth criteria and feed consumption of shrimp *Litopenaeus vannamei* fed diets enhanced with different doses of the freshwater microalgae isolate *Coelastrella terrestris* NIOF17/005.

Indices	Groups			
	D_0_	D_1_	D_2_	D_3_
FW (g)	8.46 ± 0.25^d^	9.07 ± 0.15^c^	10.23 ± 0.15^a^	9.46 ± 0.25^b^
WG (g)	6.82 ± 0.13^d^	7.41 ± 0.03^c^	8.57 ± 0.03^a^	7.80 ± 0.13^b^
DWG (g)	0.12 ± 0.00^d^	0.13 ± 0.00^c^	0.15 ± 0.01^a^	0.14 ± 0.01^b^
SGR	1.01 ± 0.03^c^	1.05 ± 0.03^bc^	1.13 ± 0.04^a^	1.08 ± 0.03^ab^
SR (%)	76.01 ± 2.00^c^	84.00 ± 2.10^b^	92.67 ± 4.16^a^	88.00 ± 2.01^ab^
FCR	2.09 ± 0.04^a^	1.94 ± 0.02^b^	1.71 ± 0.02^d^	1.83 ± 0.02^c^
FER	0.48 ± 0.03^d^	0.51 ± 0.01^c^	0.59 ± 0.01^a^	0.55 ± 0.01^b^
PER	1.33 ± 0.07^d^	1.43 ± 0.00^c^	1.63 ± 0.03^a^	1.52 ± 0.01^b^

*Note:* D_0_, D_1_, D_2_, and D_3_: diets enhanced with 0, 1, 2.5, and 5 g of the freshwater microalgae isolate *C. terrestris* NIOF17/005. Values are presented as mean ± SD (*n = 3*). Different letters in the same row indicate significant differences (*p* < 0.05).

Abbreviations: DWG, daily weight gain; FCR, feed conversion ratio; FER, feed effeciency ratio; FW, final weight; PER, protein effeciency ratio; SGR, specific growth rate; SR (%), survival rate (%); WG, weight gain.

**Table 5:** Composition analysis (%) of shrimp *Litopenaeus vannamei* fed diets supplemented with different doses of the freshwater microalgae isolate *Coelastrella terrestris* NIOF17/005.

Composition analysis (% of dry weight)	Groups			
	D_0_	D_1_	D_2_	D_3_
Dry matter	78.32 ± 0.06^b^	78.16 ± 0.18^b^	79.91 ± 0.02^a^	79.97 ± 0.09^a^
Protein	57.11 ± 0.23^c^	59.74 ± 0.12^b^	58.37 ± 0.08^a^	57.45 ± 0.24^c^
Fat	6.84 ± 0.15^b^	6.92 ± 0.08^b^	6.95 ± 0.03^b^	7.92 ± 0.43^a^
Ash	15.81 ± 0.17^b^	14.84 ± 0.15^c^	16.30 ± 0.28^a^	16.05 ± 0.36^ab^

Note: D_0_, D_1_, D_2_, and D_3_: diets supplemented with 0, 1, 2.5, and 5 g of freshwater microalgae isolate *C. terrestris* NIOF17/005. The data are presented as mean ± SD (*n = 3*). Different letters in the same row indicate significant differences (*p* < 0.05).

**Table 6:** Digestive enzyme activity, antioxidant activity, and immunological status of shrimp *Litopenaeus vannamei* fed diets supplemented with different doses of the freshwater microalgae isolate *Coelastrella terrestris* NIOF17/005.

Indices	Groups			
	D_0_	D_1_	D_2_	D_3_
Amylase (IU/L)	38.93 ± 0.94^b^	39.99 ± 0.13^b^	49.55 ± 0.17^a^	49.01 ± 0.33^a^
Lipase ( IU/L)	43.68 ± 0.20^c^	48.16 ± 3.19^b^	60.06 ± 1.18^a^	58.34 ± 1.01^a^
Catalase ( IU/g)	8.26 ± 0.41^c^	9.60 ± 0.29^b^	10.47 ± 0.22^a^	9.41 ± 0.26^b^
MDA (nmol/g)	10.74 ± 0.13^a^	10.17 ± 0.09^a^	9.99 ± 0.13^a^	10.23 ± 1.08^a^
SOD (IU/g)	9.71 ± 0.02^c^	9.66 ± 0.08^c^	10.77 ± 0.10^a^	9.91 ± 0.07^b^
Lysozyme (*µ*g/mL)	3.20 ± 0.05^c^	3.48 ± 0.24^c^	4.47 ± 0.19^a^	3.92 ± 0.18^b^

*Note:* D_0_, D_1_, D_2_, and D_3_: Diets supplemented with 0, 1, 2.5, and 5 g of the freshwater algae isolate *C. terrestris* NIOF17/005. MDA and SOD are the lipid peroxide, malondialdehyde (nmol/g), and serum superoxide dismutase (IU/g), respectively. MDA, lipid peroxide, malondialdehyde. The data are presented as mean ± SD (*n =* 3). Different letters in the same row indicate significant differences (*p* < 0.05).

Abbreviation: SOD, superoxide dismutase.

## Data Availability

The data that support the findings of this study are available from the corresponding author upon reasonable request.
